# Exercise Pretreatment Promotes Mitochondrial Dynamic Protein *OPA1* Expression after Cerebral Ischemia in Rats

**DOI:** 10.3390/ijms15034453

**Published:** 2014-03-13

**Authors:** Li Zhang, Zhijie He, Qi Zhang, Yi Wu, Xiaojiao Yang, Wenxiu Niu, Yongshan Hu, Jie Jia

**Affiliations:** 1 Department of Rehabilitation, Huashan Hospital, Fudan University, Shanghai 200040, China; E-Mails: lenzhangli@gmail.com (L.Z.); he_zhijie@hotmail.com (Z.H.); friday0451@163.com (Q.Z.); wuyi4000@163.com (Y.W.); xj8842436@163.com (X.Y.); wx_niu@126.com (W.N.); drhuys@sina.com.cn (Y.H.); 2 State Key Laboratory of Medical Neurobiology, Fudan University, Shanghai 200032, China

**Keywords:** exercise pretreatment, mitochondrial dynamics, *OPA1*, cerebral ischemia, neuroprotection

## Abstract

Exercise training is a neuroprotective strategy in cerebral ischemic injury, but the underlying mechanisms are not yet clear. In the present study, we investigated the effects of treadmill exercise pretreatment on the expression of mitochondrial dynamic proteins. We examined the expression of *OPA1*/*DLP1*/*MFF*/*Mfn1*/*Mfn2*, which regulatesmitochondrial fusion and fission, and cytochrome C oxidase subunits (*COX* subunits), which regulatemitochondrial functions, after middle cerebral artery occlusion (MCAO) in rats. T2-weighted magnetic resonance imaging (MRI) was evaluated as indices of brain edema after ischemia as well. Treadmill training pretreatment increased the expression levels of *OPA1* and *COXII/III/IV* and alleviated brain edema, indicating that exercise pretreatment provided neuroprotection in cerebral ischemic injury via the regulation of mitochondrial dynamics and functions.

## 1. Introduction

Stroke, ischemic or hemorrhagic, is considered to be a major cause of death and acquired adult disability. Ischemic brain injury can induce reactive oxygen species generation (ROS), intracellular calcium overload, signaling pathway activation, inflammation and excitotoxicity.

The prophylactic and protective effect of exercise on stroke has been addressed in clinic guidelines [1,2]. Physical activity decreases stroke incidence [3–5] and induces neuroprotection against brain damage after stroke [6,7]. In rodent ischemic models, exercise can reduce infarct volume and help in the recovery of functional behavior [8,9]. However, it is unclear how physical exercise leads to neuroprotection. Our recent studies showed that exercise induced mitochondrial transcription factors *PGC-1* and *NRF-1* expression [10] and improved mitochondrial biogenesis [11] after brain ischemic injury.

Mitochondria as a major center for cellular signaling are somehow involved in the above physiologic and pathologic conditions [12]. Neurons contain many mitochondria throughout the cytoplasm and are highly dependent on mitochondria for energy production and calcium signaling, which make them vulnerable to mitochondria dysfunction in stroke [13]. Replacing dysfunctional mitochondria with functional mitochondria might confer neuroprotection and might be a potential target for therapeutic intervention.

In many cell types, including neurons, mitochondria are highly dynamic; they move, divide, and fuse with the separation or mixing of mitochondrial proteins, lipids, and DNA/RNA. Maintaining a balance between mitochondrial fission and fusion requires several dynamin-related GTPases and proteins: mitofusin1/2 (*Mfn1*/*2*), optic dominant atrophy 1 (*OPA1*), dynamin-related protein 1 (*Drp1* or *DLP1*), mitochondrial fission factor (*MFF*) and fission protein 1 (*Fis1*) [14]. Mitochondrial fusion is mediated by Mfn1/2 and OPA1. Mfn1/2 is located in the outer mitochondrial membrane (OMM) and interacts with OPA1 located in the inner mitochondrial membrane (IMM) to couple the fusion between the OMM and IMM [15,16]. Mitochondrial fission is mediated by Drp1, MFF and Fis1. To progress to fission, Drp1 translocates from the cytosol to the OMM and interacts with MFF and Fis1 as its receptors in OMM, which leads to the fission of mitochondria [17,18].

Mitochondrial dynamics are critical for physiological energy homeostasis, calcium homeostasis, oxidative phosphorylation, reactive oxygen species management and cell death in neurodegenerative diseases [19,20]. Some studies have focused on mitochondrial biogenesis and dysfunction after ischemic stroke [21], but mitochondrial fission and fusion have not well been studied, especially in physical therapy and exercise rehabilitation for stroke. We have investigated whether exercise can affect mitochondrial dynamics in an ischemic brain model. We hypothesize that treadmill training alleviates brain damage from ischemia through the regulation of mitochondrial fusion and fission. In the present study, we investigated brain edema by magnetic resonance imaging (MRI), and analyzed the expression of mitochondrial complex proteins, mitochondrial dynamic proteins in treadmill-trained rats after ischemic injury caused by middle cerebral artery occlusion (MCAO).

## 2. Results and Discussion

### 2.1. Results

#### 2.1.1. Exercise Alleviates Brain Edema after Ischemic Injury

**Figure 1.**
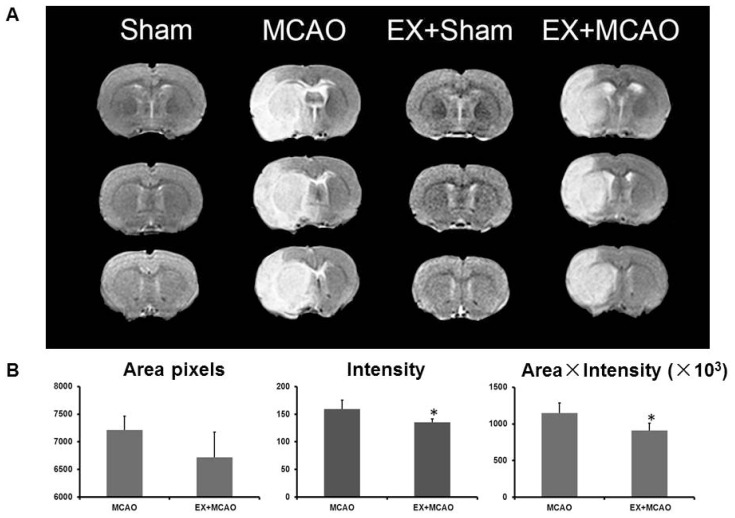
T2-weight magnetic resonance imaging (MRI) images. (**A**) The T2-weighted MRI images of series brain sections showed that significant brain edema (high signal intensity area) occurred in middle cerebral artery occlusion (MCAO) groups but not in Sham groups. Exercise pretreatment alleviated brain edema after cerebral ischemia; (**B**) The T2-weighted ischemic area and intensity were averaged ± SD and graphed. * *p* < 0.05, pre-exercised MCAO *versus* MCAO only group.

We measured the edema in ischemic brains on T2-weighted MRI images after MCAO. As shown in Figure 1A, none of the sham rats had brain edema in either the exercise-pretreatment or control groups, whereas pre-exercised ischemic brains showed significantly reduced edema compared to non-treated ischemic brains (the pre-exercised group were lower than the not-treated group by 15.1% (intensity) and 20.5% (area × intensity), *p* < 0.05, Figure 1B). These results are consistent with the data of our previous studies and other studies in the literature [22–24].

#### 2.1.2. Exercise Up-Regulates Mitochondrial COX Subunits and Mitochondria-Fusion GTPase OPA1

**Figure 2.**
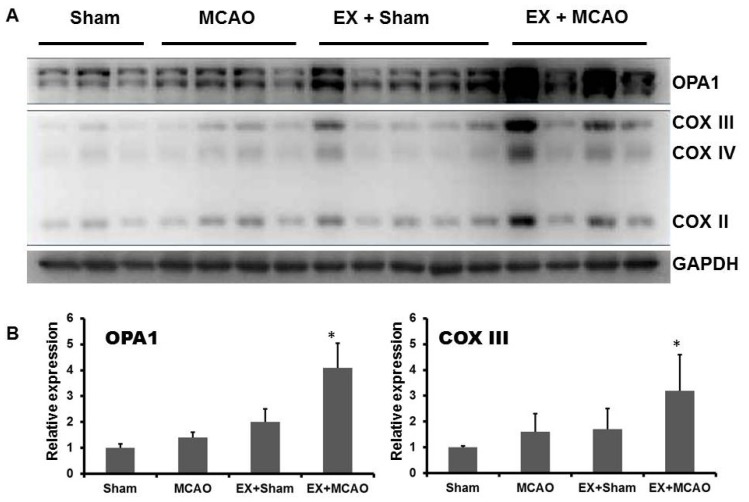
Western blot analysis of optic dominant atrophy 1 (OPA1)/C oxidase (COX)II/III/IV in the ischemic cortex. (**A**) The rats in exercise MCAO group had significantly higher levels of OPA1/COXII/III/IV; (**B**) Optical density values normalized to their respective GAPDH loading control were averaged ± SD and graphed (relative expression). * *p* < 0.05, pre-exercised MCAO *versus* MCAO only group.

To determine the effect of exercise on mitochondrial biogenesis and dynamics, we investigated mitochondrial COX II/III/IV and GTPase OPA1 (Figure 2). The level of mitochondrial complex II/III/IV significantly increased in the pre-exercised ischemia group, which shows that mitochondrial function could be improved by exercise-pretreatment even after ischemia. These data were similar to our previous study [10], in which we detected that mitochondrial complex IV increased following treadmill exercise after MCAO. In addition, the expression of the dynamin-related GTPase OPA1 increased significantly in the pretreated groups, especially in the pre-exercised ischemia group compared with the non-treated groups.

#### 2.1.3. The Effects of Exercise on Mitochondrial Dynamic Proteins in the MCAO Model

**Figure 3.**
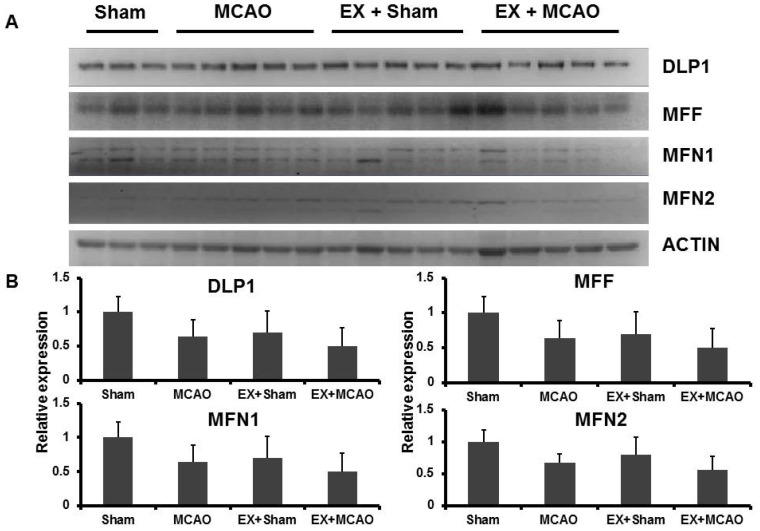
Western blot analysis of mitochondrialdynamic proteins in the ischemic cortex. (**A**) The levels of DLP1/MFF/Mfn1/2 had not significant difference between exercise and non-exercise groups; (**B**) Optical density values normalized to their respective Actin loading control were averaged ± SD and graphed (relative expression).

OPA1 induced by exercise might improve mitochondrial function in the ischemic brain by improving mitochondrial fusion. Moreover, we wondered whether other mitochondrial dynamic proteins for mitochondria fusion and fission had the relevant patterns as well. The levels of DLP1, MFF, Mfn1 and Mfn2 were determined by western blotting separately (Figure 3). Exercise-pretreatment could not significantly affect these mitochondrial dynamic proteins after ischemia.

### 2.2. Discussion

Increasing evidence supported that exercise pretreatment could be a neuroprotective strategy against ischemic stroke in either rodent or clinic studies. As our MRI data showed, exercise pretreatment alleviates brain edema at 24 h after cerebral ischemia, which is consistent with our previous study [22]. Other studies have also proved the neuroprotective effects of exercise on ameliorating blood brain barrier dysfunction, excitability from amino acid neurotoxicity and cerebral infarction volume at acute stages of stroke [23–25].

Mitochondrion, a particularly important organelle for ATP energy generation, calcium ion regulation and membrane potential maintenance, is extremely sensitive to oxidative stress during ischemia and reperfusion injury. Our present study on mitochondrial responses to ischemic injury showed that exercise pretreatment increased the levels of mitochondrial complex II/III/IV that is closely connected with cellular energy production. Simultaneously, exercise pretreatment may promote mitochondrial fusion via up-regulation of OPA1 especially after cerebral ischemia. However, in our study, the levels of other mitochondrial dynamic proteins including DLP1, MFF, Mfn1 and Mfn2 did not change in both pre-exercised and ischemic injury groups.

Mitochondrial dynamic proteins regulate mitochondrial fusion and fission through their dynamin-like GTPase activity. Promoting mitochondrial fusion and preventing mithcondrial fission in neuronal cells might protect against cell necrosis, apoptosis and neuro-degeneration after cerebral injury. A previous study revealed that the regulation of OPA1 and DLP1 in between the ischemic penumbra and the core suggested continuous mitochondrial dynamics after stroke [26]. Loss of OPA1 caused cells to release cytochrome C and leads to apoptosis [27]. The OPA1 mutant mice exhibited multi-system degeneration, which suggests that the function of OPA1 and mitochondrial dynamic proteins might be involved in aging and neuronal degeneration [28]. Moreover, inhibition of DLP1 provided the neuroprotection against both glutamate toxicity and oxygen-glucose deprivation both *in vitro* and *in vivo* [29]. Another study provided evidence that the activation of Mfn2 may protect the organelle from permeability transition that leads to programmed cell death [30]. Additionally, silencing of DLP1 inhibited mitochondrial fragmentation and cell death while silencing Mfn1/2 enhanced apoptotic death [31]. These findings suggested that mitochondrial fission and fusion plays a role in the regulation of cell apoptosis, and could be a prospective strategy for improving neuron survival and stroke outcome by regulating mitochondrial dynamic proteins.

Exercise pretreatment, or other recognized neuroprotective strategies, may have their roles in the regulation of mitochondrial biogenesis and dynamics. Our previous studies showed that post-stroke exercise treatment improved mitochondrial biogenesis [10,11]. In the present study, we found that exercise pretreatment up-regulated the level of OPA1, which may promote mitochondrial fusion. Because mitochondrial fission and fusion intertwined with each other resulting in a dynamic balance, the inhibition of fission may have the neuroprotective effect as well. Our data showed that exercise pretreatment did not alter the expression of mitochondrial fission proteins, such as DLP1, which may due to the lack of detection of phosphor-DLP1 Ser637, which is phosphorylated during DLP1 translocation to the mitochondria. Mfn1 and Mfn2 mediate outer membrane fusion, whereas OPA1 is involved in inner membrane fusion; all of three contribute to the maintenance and operation of the mitochondrial dynamic network [32]. However, in the present study, we found that exercise pretreatment increased only the expression of OPA1. A study in trained OPA1-deficient mice showed that OPA1 appeared to be necessary for the adaptive response to exercise and mitochondrial biogenesis [33]. Moreover, another study found that Mfn1/2 mRNA was progressively decreased during exercise along with decreased Mfn 1 protein levels [34]. These studies show that outer membrane and inner membrane dynamin-like GTPases Mfn1/2 and OPA1 may play different roles in exercise stress. It is emphasized that the difference could be associated with the other function of OPA1, the modeling of mitochondrial crista structures [35,36]. Otherwise, we still need to investigate the long term effect of exercise on mitochondrial fusion and fission in our further study. However, direct or convincing evidence of the regulation of mitochondrial dynamics remain unavailable because of the technical limitation of the imaging methods used to observe mitochondrial fusion and fission *in vivo*.

The rates of mitochondrial fission and fusion respond to the level of metabolism, and the pathological conditions. For example, mitochondrial fragmentation, as observed using electron microscopy, is often found in biopsies from neurodegenerative patients. More energy consumption during exercise pretreatment compared to sedentary conditions may help promote mitochondrial fusion by the regulation of mitochondrial dynamic proteins, and may maximize the capacity of stress-induced mitochondrial hyperfusion. The adaptive stress-response may induce the tolerance of a sudden energy supply shortage such as in hypoxia or cerebral vascular accidents.

The electron transport in the mitochondrial respiratory chain is the major process of reactive oxygen species (ROS) production. On the other hand, an abundance of literature indirectly supported that oxidative stress occurred during exercise in several tissues [37]. Exercise-induced oxidative stress and partial ischemia during exercise might improve the tolerance to cerebral ischemia and may provide the neuroprotective effect. Thus it will be interesting to determine the relationship between mitochondrial dynamics and ROS in further studies.

## 3. Experimental Section

### 3.1. Animals

Adult male Sprague Dawley rats, weighing 250–300 g, were purchased from the Shanghai Laboratory Animal Center of Chinese Academy of Sciences. All rats were housed in the same animal facility during a 12-h light/dark cycle with free access to food and water throughout the study. All animal experiments were approved by the Animal Experimental Committee of Fudan University (Shanghai, China) and performed in accordance with the recommendations of the National Institutes of Health Guide for the Care and Use of Laboratory Animals (Bethesda, MD, USA).

### 3.2. Exercise Pretreatment

Animals were randomly assigned to four groups: the non-exercise sham group, the exercise sham group, the non-exercise MCAO group, and the exercise MCAO group. After adaptation training, rats in the exercise groups were trained on an electronic treadmill (DSPT-202 Type 5-Lane Treadmill; Litai Biotechnology Co., Ltd., Hangzhou, China) at the speed of 20 m/min, lasting 30 min/day for 2 weeks, as described in our previous study [38]. Simultaneously, rats in the non-exercise groups remained sedentary.

### 3.3. Transient Cerebral Focal Ischemia (Transient Middle Cerebral Artery Occlusion, tMCAO)

All animal surgeries were performed without knowledge of the experimental groups. When the rats were anesthetized with chloral hydrate (360 mg/kg, i.pm), cerebral focal ischemia was induced by reversible intraluminal occlusion of the left middle cerebral artery (MCA) for 90 min as previously described [39]. Briefly, a monofilament was introduced into the internal carotid artery through the external carotid artery, advanced to the origin of the MCA, and left there for 90 min until reperfusion, which was established by the withdrawal of the monofilament. Regional cerebral blood flow (rCBF) was detected using laser Doppler flowmetry. Animals that did not show a minimum rCBF reduction of 80% or restoration to 90% of baseline were excluded. Throughout the surgery, the rectal temperature was maintained at 37.0 ± 0.5 °C with a circulating heating pad. Physiological variables, including blood pressure, heart rate and blood gases were monitored before and after surgery. In the sham control group, all of the surgical procedures were identical except for the occlusion of MCA.

### 3.4. Magnetic Resonance Imaging (MRI)

All magnetic resonance imaging (MRI) experiments were conducted on a 3T clinical MRI scanner with a maximum gradient of 45 mT/m (Siemens Trio Tim, München, Germany). Fixed in the prone position with heads in the center of a four-channel phased array rat head coil, animals were anesthetized with chloral hydrate (350 mg/kg, i.pm) during the MRI scanning. To determine the degree of brain edema after cerebral ischemia, T2-weighted MR images were acquired with the TR = 3330 ms and TE = 68 ms. For Turbo Spin Echo (TSE) acquisition, a field of view of 50 mm with an image matrix = 256 × 256, was used.

### 3.5. Western Blotting

Ipsilateral cortical tissue was harvested 3 days after MCAO. Protein extraction and Western blotting were performed as previously described [10]. Equal amounts of protein extracts were separated through 12%% SDS-polyacrylamide gel electrophoresis (SDS-PAGE) followed by transfer onto PVDF membranes. Antibodies were used for the detection of OPA1 (BD Biosciences, Franklin Lakes, NJ, USA), Mfn1 (Santa Cruz Biotechnology, Dallas, TX, USA), Mfn2 (Santa Cruz Biotechnology, Dallas, TX, USA), DLP1 (BD Biosciences, Franklin Lakes, NJ, USA), MFF (Santa Cruz Biotechnology, Dallas, TX, USA), MitoProfile OXPHOS Cocktail for COX II/III/IV (Abcam, Cambridge, MA, USA), Actin (Santa Cruz Biotechnology, Dallas, TX, USA), and GAPDH (Santa Cruz Biotechnology, Dallas, TX, USA). Band intensity (optical density) was quantified on scanned Western blot images using Quantity One software (Bio-Rad, Hercules, CA, USA) for the blots from independent experiments.

### 3.6. Statistical Analysis

Statistical analysis was performed using SPSS for Windows (SPSS Inc., Chicago, IL, USA). The quantitative data were expressed as the mean ± SEM. Parametric analysis of variance (ANOVA) was performed on the data. Unpaired *t*-tests were used for between-group comparisons of *T*2 values and Western blotting quantification. A *p*-value of less than 0.05 was considered significant.

## 4. Conclusions

In conclusion, exercise pretreatment may promote mitochondrial fusion via the up-regulation of OPA1, and this may provide the basis of neuroprotective function for against cerebral ischemia and reperfusion injury.
